# Continuous reduction in cerebral oxygenation during endurance exercise in patients with pulmonary arterial hypertension

**DOI:** 10.14814/phy2.14389

**Published:** 2020-03-18

**Authors:** Simon Malenfant, Patrice Brassard, Myriam Paquette, Olivier Le Blanc, Audrey Chouinard, Sébastien Bonnet, Steeve Provencher

**Affiliations:** ^1^ Pulmonary Hypertension and Vascular Biology Research Group Quebec City QC Canada; ^2^ Quebec Heart and Lung Institute Research Center Université Laval Quebec City QC Canada; ^3^ Department of Medicine Faculty of Medicine Université Laval Quebec City QC Canada; ^4^ Department of Kinesiology Faculty of Medicine Université Laval Quebec City QC Canada

**Keywords:** cerebral blood flow, cerebral oxygenation, cycling endurance test, exercise physiology, pulmonary arterial hypertension, pulmonary hypertension

## Abstract

**Background:**

Patients with pulmonary arterial hypertension (PAH) have lower cerebral blood flow (CBF) and oxygenation compared to healthy sedentary subjects, the latter negatively correlating with exercise capacity during incremental cycling exercise. We hypothesized that patients would also exhibit altered CBF and oxygenation during endurance exercise, which would correlate with endurance time.

**Methods:**

Resting and exercise cardiorespiratory parameters, blood velocity in the middle cerebral artery (MCAv; transcranial doppler) and cerebral oxygenation (relative changes in cerebral tissue oxygenation index (ΔcTOI) and cerebral deoxyhemoglobin (ΔcHHb); near‐infrared spectroscopy) were continuously monitored in nine PAH patients and 10 healthy‐matched controls throughout endurance exercise. Cardiac output (CO), systemic blood pressure (BP) and oxygen saturation (SpO_2_), ventilatory metrics and end‐tidal CO_2_ pressure (P_ET_CO_2_) were also assessed noninvasively.

**Results:**

Despite a lower workload and endurance oxygen consumption, similar CO and systemic BP, ΔcTOI was lower in PAH patients compared to controls (*p* < .01 for interaction). As expected during exercise, patients were characterized by an altered MCAv response to exercise, a lower P_ET_CO_2_ and SpO_2_, as wells as a higher minute‐ventilation/CO_2_ production ratio (${\dot{V}}_{E}/\dot{V}{\text{CO}}_{2}$ ratio). An uncoupling between changes in MCAv and P_ET_CO_2_ during the cycling endurance exercise was also progressively apparent in PAH patients, but absent in healthy controls. Both cHHb and ΔcTOI correlated with ${\dot{V}}_{E}/\dot{V}{\text{CO}}_{2}$ ratio (*r* = 0.50 and *r* = −0.52; both *p* < .05 respectively), but not with endurance time.

**Conclusion:**

PAH patients present an abnormal cerebrovascular profile during endurance exercise with a lower cerebral oxygenation that correlate with hyperventilation but not endurance exercise time. These findings complement the physiological characterization of the cerebral vascular responses to exercise in PAH patients.

## Graphical abstract

The novel findings of this study are that abnormal cerebrovascular responses to exercise are present during an endurance exercise protocol in patients with pulmonary arterial hypertension. It includes a reduction in cerebral oxygenation associated with higher minute‐ventilation/carbon dioxide production rather than endurance time, and an uncoupling between changes in mean blood velocity in the middle cerebral artery and end‐tidal carbon dioxide partial pressure during exercise in patients only. These findings add to the existing literature reporting abnormalities in cerebral blood flow determinants in pulmonary arterial hypertension.

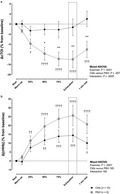

## 1 INTRODUCTION

Pulmonary arterial hypertension (PAH) is characterized by a progressive increase in pulmonary vascular resistance and a failing right ventricle (RV), resulting in early exercise intolerance and ultimately, premature death (Galie et al., 2015). Several mechanisms have been suggested to be responsible for exercise intolerance in these patients. In addition to RV dysfunction (Grunig et al., 2013), evidence of skeletal muscle and systemic vascular impairments are recognized as an important manifestation of PAH (Nickel et al., 2020). As such, it is now recognized that skeletal muscle impairments contribute to exercise intolerance, including weakness (Bauer et al., 2007; Mainguy et al., 2010), poor capillary density and oxygenation (Malenfant, Potus, Mainguy, et al., 2015; Potus et al., 2014), and poor mitochondrial oxidative phosphorylation and metabolism (Batt, Ahmed, Correa, Bain, & Granton, 2014; Malenfant, Potus, Fournier, et al., 2015). Recent studies have also proposed impaired cerebral blood flow (CBF) (Malenfant et al., 2017) and oxygenation (Malenfant et al., 2017; Muller‐Mottet et al., 2015) as limiting factors for maximal exercise capacity in these patients.

Most PAH patients’ complaints refer to exertional dyspnea. While the neurophysiology of dyspnea has yet to be explained in PAH, several lines of evidence suggest that lower prefrontal oxygenation recently described in these patients during incremental exercise testing (Malenfant et al., 2017; Muller‐Mottet et al., 2015) could be involved. Indeed, based on the multichannel near‐infrared spectroscopy (NIRS) technique, Higashimoto et al. demonstrated that exertional dyspnea was associated with both prefrontal and premotor cortex overactivation in chronic obstructive pulmonary disease (COPD) patients (Higashimoto et al., 2011, 2015). Prefrontal oxygenation during incremental exercise to exhaustion also correlated with exercise capacity in PAH (Malenfant et al., 2017). However, dyspnea is most commonly reported at submaximal exercise level in PAH (Mainguy, Provencher, Maltais, Malenfant, & Saey, 2011). More importantly, cycling endurance tests were shown to elicit a markedly different physiological response compared to incremental cycling exercise to exhaustion in PAH (Mainguy et al., 2014). Thus, whether a submaximal exercise induces similar cerebrovascular and prefrontal oxygenation changes compared to incremental exercise to exhaustion in PAH remains to be explored.

Therefore, the objective of the current study was to assess changes in cerebral oxygenation and its physiological determinants during an endurance cycling protocol to further understand the cerebrovascular contribution to exercise intolerance in PAH patients compared to healthy controls. We hypothesized that patients would exhibit altered MCAv and oxygenation during endurance exercise, correlating with total endurance time.

## 2 METHODS

### 2.1 Ethics and informed consent

The *Comité d’éthique de la recherche de l’IUCPQ‐Université Laval* (CER: 20975) approved the study according to the principles established in the Declaration of Helsinki (except for registration in a database). Informed consent was obtained by all participants prior to the investigation.

### 2.2 Participants

A total of nine World Health Organization (WHO) class II‐III idiopathic or hereditary PAH patients completed the study. PAH was defined as mean pulmonary artery pressure ≥25 mmHg, with a pulmonary capillary wedge pressure ≤15 mmHg (Galie et al., 2015). Only patients with stable condition for the past 4 months were eligible. Exclusion criteria were as follows: 6MWT distance <300 m; left ventricular ejection fraction <40%; restrictive lung disease evidenced as lung fibrosis on computed tomography scan or total lung function <80% of predicted value; obstructive lung disease evidenced as forced expiratory volume in the first second of expiration/forced vital capacity ratio <70%; body mass index >30 kg.m^−2^; presence of a metabolic disease. Ten healthy sedentary participants individually matched for age and sex were recruited to serve as control participants. All measurements were performed in a thermoneutral laboratory over one visit. All PAH women in reproductive age were taking oral contraceptives continuously or had an intrauterine device in accordance with current guideline regarding pregnancy and PAH (Galie et al., 2015). Care was taken to recruit healthy women using similar contraceptive measures as their paired PAH counterpart. Although this study was part of a larger study that examined the influence of PAH on key determinants and exercise‐induced changes in CBF and oxygenation at rest and during an incremental cycling protocol to exhaustion (Malenfant et al., 2017), the current study addressed an experimental question determined a priori. Participants were instructed to refrain from exercise, alcohol, caffeine consumption, and heavy meal 12 hr prior to experimental testing.

### 2.3 Study design

#### 2.3.1 Endurance protocol

Participants reported to the laboratory 48 hr after completion of the main protocol (refer to [Malenfant et al., 2017]) to perform a cycling endurance test on an electromagnetically braked ergocycle (Lode BV Corival, Groningen, The Netherlands) according to the current guidelines (American Thoracic & American College of Chest, 2003). Following a 5‐min rest period and 1 min of unloaded pedaling, the endurance cycling protocol was initiated at 75% of the maximal work rate achieved during the incremental cardiopulmonary exercise test (Malenfant et al., 2017). Patients were instructed to cycle at a minimum cadence of 60 rpm. Standardized encouragement was provided throughout the endurance cycling protocol. The endurance time represented the total exercise duration after workload increase until exhaustion, defined as the inability to maintain a minimum cadence of 60 rpm.

#### 2.3.2 MCAv, oxygenation and physiological measurements

Heart rate (HR) was measured with cardiac 12‐lead ECG. Minute‐ventilation (${\dot{V}}_{E}$), oxygen consumption (${\dot{V}}_{E}/\dot{V}O_{2}$), and carbon dioxide production (${\dot{V}}_{E}$), oxygen consumption (${\dot{V}}_{E}/\dot{V}{\text{CO}}_{2}$) were continuously monitored through a breath‐by‐breath expired gas analyzer (Breeze Suite, MedGraphics Corp.). Systemic oxygen saturation (SpO_2_) was continuously measured by pulse oximetry (Nonin Medical Inc). Mean arterial pressure (MAP) and cardiac output (CO) were both measured noninvasively by the Modelflow finger photoplethysmography technique (Nexfin, Edwards Lifesciences). This technique proves reliable to evaluate absolute CO at rest and during exercise in PAH patients (Lador et al., 2015). The cuff was placed on the left middle finger and referenced to the level of the heart using a height correcting unit. MCAv was monitored with transcranial Doppler ultrasonography (2.0‐MHz probe; Doppler Box Compumedics DWL USA, Inc.). Identification and location of the left MCA was obtained using standardized procedures (Willie et al., 2011). The probe was attached to a commercially available headset and maintained in position using adhesive conductive ultrasonic gel (Tensive, Parker Laboratory) to ensure a stable position and angle throughout the endurance cycling protocol. Cerebral oxygenation was monitored by NIRS in the prefrontal cortex capillary beds using a single‐distance, continuous wave light, dual‐channel Oxiplex TS (ISS). Relative changes (Δ) from baseline in cerebral oxygenated (ΔcHbO_2_), deoxygenated (ΔcHHb), and total (ΔcHb_tot_) hemoglobin concentration were measured. The NIRS fiber optode consisted of eight light‐emitting diodes operating at wavelengths of 690 and 830 nm and one detector fiber bundle with a separated distance of approximately 4 cm, corresponding to a light penetration depth of 2 cm in the brain tissue. The signal was analyzed using the modified Beer–Lambert law. Metrics evaluated were [cHbO_2_]/[cHb_tot_] ratio, expressed as Δ from baseline in cerebral tissue oxygenation index (%ΔcTOI), while %ΔcHHb was used as a surrogate of the local oxygen delivery and utilization matching.

#### 2.3.3 Data acquisition and analysis

For MCAv, CO, MAP, and cerebral oxygenation metrics (% ΔcHHb and % ΔcTOI), signals were acquired at 1,000 Hz via an analog‐to‐digital converter (Powerlab 16/30 ML880; ADInstruments) and stored offline for subsequent analysis using commercially available software (LabChart version 7; ADInstruments). Breath‐by‐breath respired gas and SpO_2_ were simultaneously acquired and stored offline for subsequent analysis. Changes in cerebral oxygen delivery from baseline during exercise was estimated according to the following formula: ΔcDO_2_ = ΔMCAv × Δarterial oxygen content (estimated as 1.349 × hemoglobin × pulse oximetry (SpO_2_)). Exercise data from finger photoplethysmography and transcranial Doppler ultrasonography were resampled at 1 Hz and time‐aligned with breath‐by‐breath measurements. Data points (MCAv, cDO_2_, SpO_2_, P_ET_CO_2_, ΔcHHb, ΔcTOI, ${\dot{V}}_{E}/\dot{V}O_{2}$, CO, HR, MAP, ${\dot{V}}_{E}$, ${\dot{V}}_{E}/\dot{V}{\text{CO}}_{2}$ ratio) were analyzed at different stages of exercise (rest, warm‐up, 25%, 50%, 75% of total exercise time, exercise exhaustion, and 1‐min postexercise cooldown). Each time point value corresponded to a 10‐s average value.

### 2.4 Statistical analysis

Data are reported as mean ± standard deviation unless specified. Unpaired *t* tests were used to compare variables from both groups at baseline when data were normally distributed. Normality was assessed using D’Agostino and Pearson normality tests. When not normally distributed, data were transformed using log_10_. Unpaired *t* tests were then applied to transformed data. For exercise data, a mixed ANOVA (between‐subjects factor: group; within‐subjects factor: endurance time) was used. After a positive identification of an interaction effect (endurance time x groups), differences were located using paired (within‐group) and independent (between‐group) samples *t* tests, with Bonferroni correction. Pearson correlation coefficient was used to evaluate the relationship between ΔcHHb and ΔcTOI and exercise determinants unless otherwise specified. Power analysis indicated that eight patients would be needed to achieve a α = 0.05 and a β = 0.80 for a difference of 13 cm/s and −8.0 ± 6.0% in the main study outcome (resting MCAv and exercise cTOI, respectively; using G*Power v3.1.9.3). A total of 11 patients were recruited for the larger protocol (Malenfant et al., 2017) to account for potential dropouts. Statistical significance was accepted at *p* < .05.

## 3 RESULTS

Baseline characteristics of PAH patients and control participants are presented in Table 1. Groups were similar for sex, age, and body mass index. Most patients were in WHO functional class II on combination therapy. Patients achieved 89% of their predicted walking distance for the 6MWT. Endurance time was 36% lower in PAH patients compared to healthy participants.

**TABLE 1:** Baseline characteristics

	PAH group *n* = 9	Control group *n* = 10
Sex, M/F	3/6	3/7
Age, years	45 (12)	44 (15)
BMI, kg m^−2^	25.0 (4.1)	25.5 (3.1)
PAH subtype		
iPAH‐HPAH ratio	7/2	NA
WHO functional class		
II/III	8/1	NA
Resting hemodynamics		
mPAP, mmHg	47 (10)	NA
CI, L min^−1^ m^−2^	3.0 (0.5)	NA
PVR, dyne s^−1^ cm^−5^	568 (187)	NA
PAOP, mmHg	10 (2)	NA
SvO_2_, %	70 (4)	NA
PAH‐targeted agents		
Prostacyclin analogue	1	NA
PDE‐5i	8	NA
ERA	5	NA
Monotherapy	4	NA
Combination therapy	5	NA
Exercise capacity		
6‐Min walk test, m	515 (93)	NA
% predicted	89 (22)	NA
Endurance exercise		
75% WR, Watts	69 (26)	121 (43)**
Time, s	480 (266)	755 (326)*

Note

Data are presented as mean (standard deviation) unless otherwise specified.

Abbreviations: BMI: body mass index; CI: cardiac index; CPET: cardiopulmonary exercise test; ERA: endothelin receptor antagonist; mPAP: mean arterial pressure; PAH: pulmonary arterial hypertension; PAOP: pulmonary artery occlusion pressure; PDE‐5i: phosphodiesterase type 5 inhibitors; PVR: pulmonary vascular resistance; RER: respiratory equivalent ratio; SvO_2_: systemic venous oxygen saturation; V_E_/VCO_2_: ventilatory equivalent for CO_2_ slope; VO_2_: oxygen consumption; WR: work rate.

Significant difference between PAH and control groups: *p < .05; **p < .01; ***p < .001; ****p < .0001.

**Figure 1:**
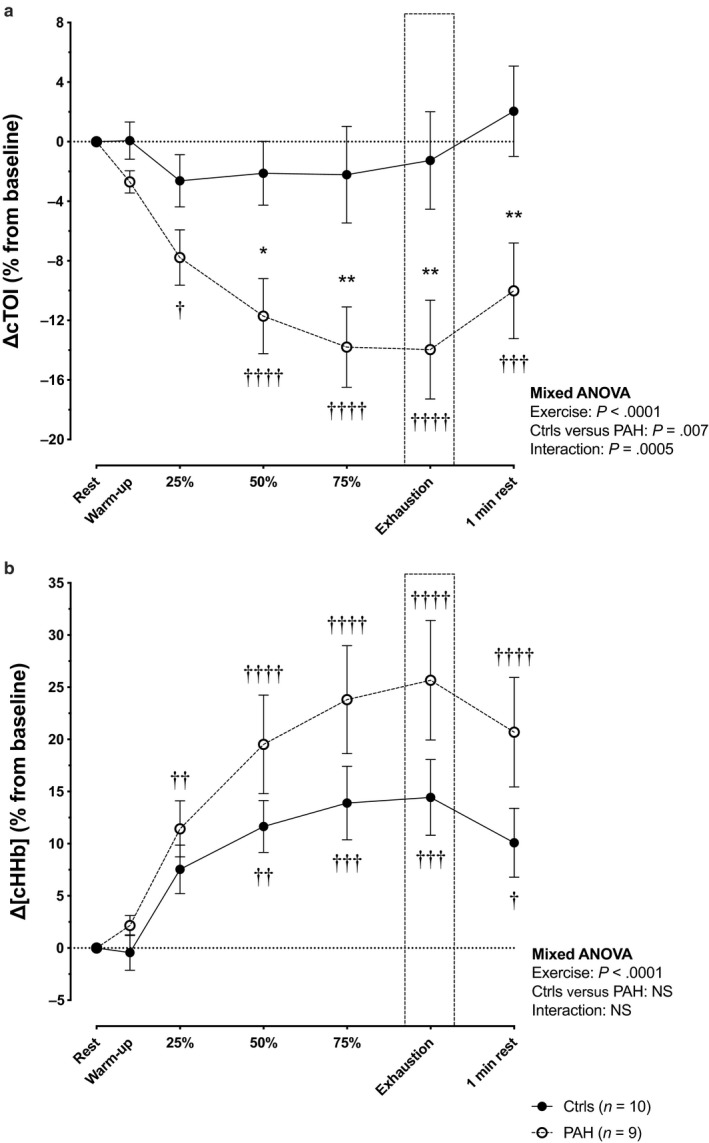
(a) Changes in cerebral tissue oxygenation index (ΔcTOI); (b) Changes in cerebral deoxyhemoglobin (ΔcHHb) in pulmonary arterial hypertension patients (PAH: open circles) and controls (Ctrls: closed circles) during cycle endurance test. The x‐axis represents the different stage of exercise (rest, warm‐up, 25%, 50%, 75% of total exercise time, exercise exhaustion, and 1‐min rest). The unloaded pedaling is the average of the last 30 s before workload onset. Significant difference from baseline: †*p* < .05; ††*p* < .01; †††*p* < .001; ††††*p* < .0001. Significant difference between Ctrls and PAH groups: *p < .05; **p < .01

**Figure 2:**
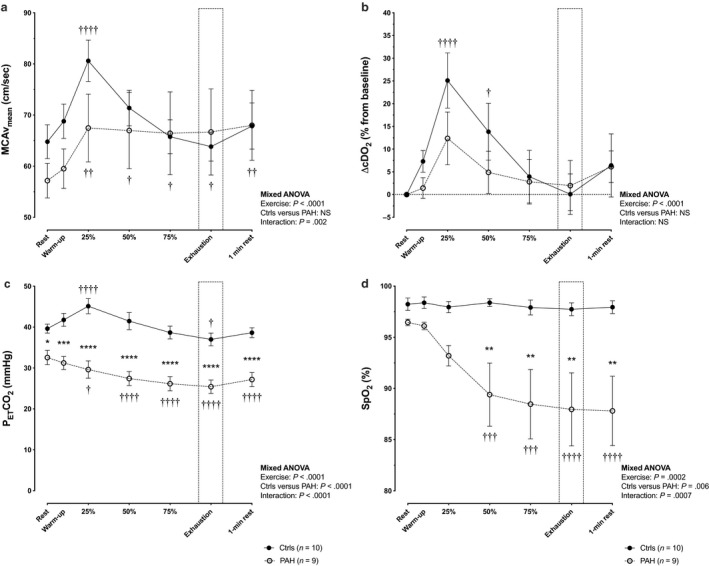
(a) Mean blood flow velocity in the middle cerebral artery (MCAv); (b) Changes in estimated cerebral oxygen delivery (ΔcDO_2_); (c) Systemic oxygenation response (SpO_2_); (d) End‐tidal CO_2_ pressure (P_ET_CO_2_) in pulmonary arterial hypertension patients (PAH: open circles) and controls (Ctrls: closed circles) during cycle endurance test. The x‐axis represents the different stage of exercise (rest, warm‐up, 25%, 50%, 75% of total exercise time, exercise exhaustion, and 1‐min rest). The unloaded pedaling is the average of the last 30 s before workload onset. Significant difference from baseline: †*p* < .05; ††*p* < .01; †††*p* < .001; ††††*p* < .0001. Significant difference between Ctrls and PAH groups: *p < .05; **p < .01; ***p < .001; ****p < .0001

PAH patients had a constant decrease in ΔcTOI throughout the endurance cycling protocol, whereas it remained unchanged in controls (*p* = .0005 for interaction; Figure 1a). Consistently, ΔcHHb increased more prominently in PAH patients, although the difference did not reach statistical significance (*p* = .14 for interaction; Figure 1b). Despite higher ${\dot{V}}_{E}/\dot{V}{\text{CO}}_{2}$ ratio (Table 2) and lower P_ET_CO_2_ (Figure 2c) compared to healthy controls, exercise‐induced changes in MCAv (Figure 2a), systemic blood pressure, CO (Table 2), and ΔcDO_2_ (Figure 2b) were comparable between groups, and thus could not entirely explain the impaired cerebral oxygenation. Indeed, while controls initially exhibited a quick surge in MCAv and ΔcDO_2_, subsequent changes were similar between groups. Conversely, PAH patients displayed lower V̇O_2_ and SpO_2_ throughout the exercise protocol (Table 2; Figure 2d).

**TABLE 2:** Endurance cycling exercise at 75% of peak workload

	PAH group *n* = 9	Control group *n* = 10	Mixed ANOVA
$\dot{V}$˙O_2_, mlO_2_ min^−1^ Kg^−1^			
Rest	4.1 (0.7)	3.6 (0.4)	Exercise *p* < .0001 Ctrls Vs. PAH *p* = .0008 Interaction *p* < .0001
Warm‐up	6.9 (1.1)	7.2 (1.6)†	
25%	14.7 (4.0)††††	24.3 (4.7)††††,****	
50%	16.5 (4.0)††††	26.8 (5.7)††††,****	
75%	17.6 (4.4)††††	28.0 (6.4)††††,****	
Exhaustion	18.0 (4.4)††††	28.1 (6.1)††††,****	
1‐Min rest	14.4 (2.3)††††	16.9 (2.7)††††	
CO, L min^−1^			
Rest	5.6 (1.4)	5.7 (1.5)	Exercise *p* < .0001 Ctrls Vs. PAH NS Interaction NS
Warm‐up	7.5 (2.2)	7.5 (2.1)	
25%	9.7 (3.0)††††	11.6 (5.0)††††	
50%	10.6 (3.7)††††	12.5 (5.2)††††	
75%	10.7 (3.6)††††	12.6 (5.0)††††	
Exhaustion	11.1 (3.5)††††	12.2 (5.4)††††	
1‐Min rest	9.7 (3.4)††††	10.3 (4.5)††††	
HR, bmp			
Rest	71 (11)	76 (13)	Exercise *p* < .0001 Ctrls Vs. PAH *p* = .04 Interaction *p* = .02
Warm‐up	87 (10)†††	96 (12)††††	
25%	122 (18)††††	144 (19)††††,*	
50%	135 (18)††††	154 (20)††††	
75%	140 (16)††††	159 (21)††††	
Exhaustion	142 (15)††††	161 (22)††††	
1‐Min rest	123 (15)††††	138 (20)††††	
MAP, mmHg			
Rest	96 (14)	99 (9)	Exercise *p* < .0001 Ctrls Vs. PAH NS Interaction NS
Warm‐up	104 (20)	100 (13)	
25%	131 (31)††††	130 (19)††††	
50%	135 (30)††††	129 (14)††††	
75%	137 (31)††††	122 (12)†††	
Exhaustion	132 (33)††††	122 (8)†††	
1‐Min rest	119 (26)†††	102 (11)	
${\dot{V}}_{E}$, L min^−1^			
Rest	12 (3)	8 (1)	Exercise *p* < .0001 Ctrls Vs. PAH NS Interaction NS
Warm‐up	20 (4)	14 (4)	
25%	50 (23)††††	53 (13)††††	
50%	61 (24)††††	63 (15)††††	
75%	69 (30)††††	68 (21)††††	
Exhaustion	72 (31)††††	72 (26)††††	
1‐Min rest	53 (20)††††	49 (16)††††	
${\dot{V}}_{E}/\dot{V}{\text{CO}}_{2}$ ratio			
Rest	44 (6)	35 (3)**	Exercise *p* < .0001 Ctrls Vs. PAH *p* < .0001 Interaction *p* = .0003
Warm‐up	43 (6)	31 (3)†,***	
25%	43 (7)	26 (3)††††,****	
50%	46 (9)	29 (5)†††,****	
75%	49 (10)†	31 (4)****	
Exhaustion	50 (10)†††	33 (5)****	
1‐Min rest	47 (9)	31 (3)****	

Note

Data are presented as mean (standard deviation) unless otherwise specified.

Abbreviations: CO: cardiac output; HR: heart rate; MAP: mean arterial pressure ${\dot{V}}_{E}$: minute ventilation; ${\dot{V}}_{E}$/${\dot{V}}_{E}$CO_2_ ratio: ventilatory equivalent for CO_2_; $\dot{V}$˙O_2_: oxygen consumption.

Significant difference from baseline: †*p* < .05; ††*p* < .01; ††† *p* < .001; ††††*p* < .0001.

Significant difference between Ctrls and PAH groups: *p < .05; **p < .01; ***p < .001; ****p < .0001.

Exploration of the potential mechanisms associated with changes in end‐exercise cerebral oxygenation indicated a mild correlation between end‐exercise ${\dot{V}}_{E}/\dot{V}{\text{CO}}_{2}$ ratio and both ΔcHHb and ΔcTOI, while ΔcHHb correlated with end‐exercise SpO_2_ (Table 3). No correlation was found between cerebral oxygenation metrics and endurance time. Of note, MCAv remained elevated in patients after its initial surge until 1‐min postexercise (Figure 2a), notwithstanding a constant P_ET_CO_2_ reduction (Figure 2d). This pattern suggests an uncoupling between MCAv and P_ET_CO_2_ in patients only.

**TABLE 3:** Correlations between exercise tolerance determinants and cerebral oxygenation

	cTOI, % change from baseline		cHHb, % change from baseline	
	Pearson *r*	*p* value	Pearson *r*	*p* value
End‐exercise ${\dot{V}}_{E}$˙O_2_, mlO_2_ min^−1^ kg^−1^	.25	.29	−.06	.78
End‐exercise CO, L/min	.10	.73	<.01	.99
End‐exercise MCAv _mean_, cm/s	−.18	.45	.35	.15
End‐exercise ${\dot{V}}_{E}/\dot{V}{\text{CO}}_{2}$	−.50	**.03**	.52	**.02**
End‐exercise P_ET_CO_2_, mmHg	.44	.06	−.44	.06
End‐exercise SpO_2_, %	.49	.06	−.53	**.03**
^a^Endurance time, s	.30	.22	−.12	.63

Abbreviations: 6MWT: 6 min walk test; cHHb: cerebral deoxyhemoglobin at exhaustion; cTOI: cerebral tissue oxygenation index at exhaustion; mPAP: mean pulmonary arterial pressure; PVR: pulmonary vascular resistance; SvO_2_: systemic venous oxygen saturation; ${\dot{V}}_{E}$/${\dot{V}}_{E}$CO_2_: ventilatory equivalent for CO_2_ slope.

^a^ Spearman r.

## 4 DISCUSSION

To our knowledge, this is the first study to compare MCAv and cerebral oxygenation and their physiological responses to cycling endurance exercise in sex‐ and age‐matched PAH patients and healthy controls. Our principal finding was that PAH patients displayed lower cerebral oxygenation throughout the endurance cycling protocol compared to healthy controls. Furthermore, those changes were not correlated with endurance time, but were mildly correlated with a steeper rise in ${\dot{V}}_{E}/\dot{V}{\text{CO}}_{2}$ ratio and SpO_2_. Lastly, patients presented an uncoupling between MCAv and P_ET_CO_2_ throughout the exercise protocol. Altogether, the present study results indicate that the cerebrovascular response to cycling endurance exercise in PAH differs from incremental cycling exercise to exhaustion, thus complementing the physiological characterization of the MCAv and cerebral oxygenation responses to exercise in PAH patients.

### 4.1 Cerebral oxygenation and ventilatory response to exercise in PAH

During incremental exercise, PAH patients are characterized by an exaggerated ventilatory response to exercise, as expressed by a disproportionate rise in ${\dot{V}}_{E}/\dot{V}{\text{CO}}_{2}$ ratio, hypocapnia and hypoxemia, consistent with ventilation/perfusion mismatch (Deboeck, Niset, Vachiery, Moraine, & Naeije, 2005; Sun, Hansen, Oudiz, & Wasserman, 2001; Vicenzi et al., 2016; Yasunobu, Oudiz, Sun, Hansen, & Wasserman, 2005). The pattern of endurance exercise response in our patients is consistent with those findings. A possible association between low cerebral oxygenation and exercise‐induced hyperventilation may be suggested by the correlation found between markers of cerebral oxygenation, for example ΔcHHb and ΔcTOI, and end‐exercise ${\dot{V}}_{E}/\dot{V}{\text{CO}}_{2}$ ratio and SpO_2_, suggesting that low brain O_2_ saturation might stimulate excessive ventilation in PAH.

Potential explanations for this finding might reside in three physiological mechanisms: (a) a potential brain O_2_ sensor, (b) overstimulation and hyperadditivity of central and peripheral chemoreceptors, and (c) sensory feedback from group III/IV skeletal muscle afferents. First, a commonly held view is that the central nervous system lacks a physiological O_2_ sensor capable of stimulating the brain stem respiratory network and lung ventilation in response to decrease in O_2_ delivery and saturation (Gourine & Funk, 2017; Neubauer & Sunderram, 2004). However, recent neuroscience advances in understanding the O_2_ sensing capability described that brain hypoxia sensors might be found in the brain stem astrocytes (Gourine et al., 2010). When activated in hypoxic conditions, brain stem ectonucleotidase activity leads to rapid degradation of adenosine triphosphate, which stimulates brain presympathetic neurons in the central respiratory circuit leading to increases in lung ventilation (Gourine et al., 2010; Rajani et al., 2018). Active neurons can adapt to limited O_2_ delivery and metabolic stress by dilating nearby arterioles, and capillaries by adapting pericytes contractility (Hall et al., 2014; Kisler et al., 2017) and rising intracellular calcium in astrocytes (Mishra et al., 2016). As such, brain hypoxia sensors can modulate the neurovascular coupling to regional cerebral deoxygenation via astrocytes and pericytes, which in turn stimulate the brain stem neuronal respiratory network resulting in hyperventilation. Secondly, both peripheral and central chemoreceptors overactivity in PAH (Malenfant et al., 2017; Paula‐Ribeiro et al., 2019; Vicenzi et al., 2016) might stimulate ventilation in an additive manner. Smith et al. demonstrated an hyperadditive two to fourfold response change in the central chemoreceptor response to carotid chemoreceptors hypersensitivity, resulting in further increase in ventilation in awaked dogs (Smith, Blain, Henderson, & Dempsey, 2015). Lastly, a potential‐increased sensory feedback from group III/IV skeletal muscle afferents might further increase ventilatory response to exercise, thus contributing to a further decrease in P_ET_CO_2_ and ultimately blunting the MCAv increase response to exercise (Braz et al., 2014). Nonetheless interesting, those mechanisms remain to be confirmed in PAH animal models and remain speculative.

### 4.2 Cerebral oxygenation and endurance time

Based on findings from our recent study that documented a correlation between lower cerebral oxygenation and exercise capacity during incremental testing in PAH patients (Malenfant et al., 2017), it was reasoned that endurance cycling would also display an association with exercise intolerance. However, cerebral oxygenation was not associated with endurance time. Two outcomes of this study might explain this lack of association. First, we previously documented an attenuated dynamic cerebral autoregulation in PAH and as such, changes in MAP are more passively transmitted to the brain. The rather normal CO and MAP responses to endurance exercise should have resulted in a parallel increase in MCAv in patients. However, MCAv only mildly increased compared to healthy controls. A potential explanation for these equivocal findings is that a reduction in P_ET_CO_2_ occurs secondary to hyperventilation resulting in a subsequent cerebral vasoconstriction (Braz et al., 2014) and leading to lower ΔcHHb and ΔcTOI. However, prefrontal cortical oxygenation may not be representative of changes in local capillary and/or venous oxygenation in the brain. Therefore, endurance time may not be sensitive enough in this context to demonstrate an association with cerebral oxygenation metrics. Secondly, cerebral O_2_ delivery at the different stages of exercise or at exhaustion was not different between groups despite longer endurance time in healthy controls. Therefore, despite a relatively normal O_2_ delivery at exercise, this lowered cerebral oxygenation might indicate O_2_ redistribution to active neurons in the motor cortex at the cost of the frontal cortex where cerebral oxygenation was monitored. Hence, cerebral oxygenation accounts as a limiting factor for exercise intolerance (Malenfant et al., 2017), but most likely through increased exercise hyperventilation. As demonstrated in COPD (Vogiatzis et al., 2013) and more recently in PAH (Ulrich et al., 2017), O_2_ supplementation increases endurance time but not cerebral O_2_ delivery, indicating that the supplementation most probably alleviates dyspnea sensation and legs discomfort to allow longer endurance time without normalizing either cerebral or skeletal muscle O_2_ delivery (Malenfant, Potus, Mainguy, et al., 2015).

### 4.3 Uncoupling between MCAv and P_ET_CO_2_ during endurance exercise in PAH and potential clinical consequences

Another illustration of cerebrovascular abnormalities might reside in the observation of an uncoupling between changes in MCAv and P_ET_CO_2_ during the endurance exercise protocol, for example, absence of decrease in cerebral perfusion notwithstanding hyperventilation‐induced hypocapnia, apparent in PAH patients, but absent in healthy controls. This finding has been previously observed in athletes with post‐concussion syndrome (Clausen, Pendergast, Willer, & Leddy, 2016; Imhoff et al., 2017). Hence, alterations in two key CBF regulation mechanisms in PAH, for example, an attenuated dynamic cerebral autoregulation and a lower cerebrovascular reactivity to CO_2_, might result in the inability of exercise‐induced hypocapnia to dampen changes in MCAv. As such, CBF becomes more reliant on MAP (Malenfant et al., 2017; Treptow et al., 2016), and may eventually lead to a progressive breakdown of the blood–brain barrier resulting in extravascular edema and cerebral vessel frailty (Ogoh & Ainslie, 2009). Whether these abnormal CBF regulation mechanisms contribute to a 1.5 times higher odds of developing a stroke for PAH patients remains to be addressed (Shah, Sutaria, & Vyas, 2019).

### 4.4 Limitations

The validity of MCAv as a surrogate of CBF encompasses minimal variation in MCA diameter across a variety of physiological challenges. However, the MCA diameter is changing homogeneously among participants in response to changes in P_ET_CO_2_ (Coverdale, Gati, Opalevych, Perrotta, & Shoemaker, 2014; Verbree et al., 2014). Also, in response to handgrip exercise, a MCA diameter vasoconstriction of 2% was observed, without change in P_ET_CO_2_ (Verbree et al., 2017). Altogether, these results indicate that the MCA is vasoactive under P_ET_CO_2_ changes and during exercise. In our patients, P_ET_CO_2_ dropped by ~7 mmHg at the end of the endurance exercise (corresponding to ~2% MCA vasoconstriction). Based on the model developed by Ainslie and Hoiland, a 2% MCA vasoconstriction is likely to result in a <5% discrepancy between flow and velocity (Ainslie & Hoiland, 2014). Hence, MCAv may be considered as a CBF surrogate for our patients. However, we acknowledge limitations of this interpretation. First, the aforementioned studies were conducted only in healthy subjects and secondly, other factors might influence the MCA diameter in PAH patients such as a lower cerebrovascular reactivity to CO_2_ (Malenfant et al., 2017; Treptow et al., 2016), attenuating the influence of hypocapnia on MCA diameter during exercise. Therefore, we recognize that care must be taken when interpreting the present data about MCAv as a surrogate of CBF in the present study.

Changes in skin blood flow may influence absolute NIRS light absorption (Miyazawa et al., 2013). We reported relative changes in percentage of cerebral TOI and HHb to minimize this confounding factor. Moreover, increase in cHHb is a known representative of altered O_2_ delivery in the cerebral capillary bed and is expected to be associated with impaired cerebral oxygen extraction.

The vascular O_2_ content depends on a balance between CBF and cerebral metabolic rate of oxygen, the latter depending on the O_2_ pool, the blood–brain barrier integrity, and the hemoglobin dissociation curve (Valabregue, Aubert, Burger, Bittoun, & Costalat, 2003). We acknowledge that hypocapnia and early lactic acid accumulation during exercise might potentially add to the burden of lower cerebral oxygenation because of an early rightward and downward shift of the hemoglobin dissociation curve, decreasing O_2_ affinity for Hb and therefore facilitating its release in the cerebrovascular circulation, which can influence the O_2_ content at the capillary end and contributing to its heterogeneity.

No significant changes were found in the MCAv of six healthy male subjects after 30 min and 60 min following administration of 100 mg of sildenafil (Arnavaz et al., 2003), while in a separate study, administration of the same dose of sildenafil in ten healthy controls causes no significant change in MCAv and artery diameter, but was associated with higher prevalence of headaches (Kruuse, Thomsen, Jacobsen, & Olesen, 2002). As such, the impact of sildenafil on the MCAv remains unlikely for our patients. However, a potential influence of PAH therapies cannot be excluded as sildenafil displayed beneficial effects on cerebral vascular reactivity indicative of an improvement in neurovascular coupling when investigated, while inhaled iloprost provoked deterioration of cerebral microvascular tone and oscillating properties in the posterior cerebral arteries of the same patients (Rosengarten et al., 2006). Therefore, a class‐effect may not be ruled out.

Lastly, the discrepancy between P_ET_CO_2_ and PaCO_2_ in PAH must be discussed. In contrast to healthy subjects in whom the arterial to end‐tidal CO_2_ pressure gradient (Pa – _ET_CO_2_) is reduced and close to zero during maximal exercise when corrected for temperature (Losa‐Reyna, Torres‐Peralta, Henriquez, & Calbet, 2015), this gradient remains slightly positive in PAH (Sun, Hansen, Oudiz, & Wasserman, 2002) suggesting an increased physiological dead space fraction. Surprisingly, however, a seminal study comparing lung absorption of multiple inert gases showed no or a limited increase in V_D_/V_T_ despite marked pulmonary arterial obstruction (Dantzker & Bower, 1979). Importantly, both P_ET_CO_2_ and PaCO_2_ are physiologically reduced at rest and during exercise in proportion to disease severity (Yasunobu et al., 2005) and independently predict mortality (Hoeper, Pletz, Golpon, & Welte, 2007). Moreover, both metrics exhibit parallel changes during exercise in pulmonary hypertension and accordingly, P_ET_CO_2_ is considered an imperfect although a relevant surrogate for PaCO_2_ in PAH (Neder et al., 2016).

## 5 CONCLUSION

The present study provides physiological evidence that PAH patients have functional abnormalities in MCAv and cerebral oxygenation during an endurance exercise test. These alterations include a reduction in cerebral oxygenation associated with end‐exercise ${\dot{V}}_{E}/\dot{V}{\text{CO}}_{2}$ ratio and SpO_2_ and an uncoupling between changes in MCAv and P_ET_CO_2_ in patients only. Altogether, these findings indicate a different cerebrovascular response to exercise between incremental and endurance exercise in PAH.

## CONFLICT OF INTEREST

The authors have declared no conflict of interest.
